# New Methods in Food Processing and Analysis

**DOI:** 10.3390/foods14213669

**Published:** 2025-10-28

**Authors:** Marc Regier

**Affiliations:** Food Process Engineering, Trier University of Applied Sciences, 54293 Trier, Germany; regier@hochschule-trier.de; Tel.: +49-651-8103-296

The food industry is currently undergoing a profound transformation. Growing demands for sustainable production; heightened expectations of safety, quality, and quantity; and the urgent need for greater efficiency call for innovative solutions (see, for example, [1] and the references therein). The transformation of the food system is supported and simultaneously demanded by the UN Sustainable Development Goals [2] and related initiatives such as the Global Panel [3]. An important task in this regard is assigned to production, processing and analysis [4].

This Special Issue on *New Methods in Food Processing and Analysis* brings together research that highlights how new technologies can help shape the future of food, addressing some of these demands.

The articles in this collection present advances that, in part, go beyond incremental improvements. They showcase novel processing technologies, ranging from ultrasound to pulsed electric fields as pretreatment for innovative extraction processes and drying to ethanol pickling.

At the same time, state-of-the-art analytical tools, such as laser-induced breakdown and fluorescence spectroscopy (combined with machine learning) for olive oil authentication and real-time-monitoring of dough mixing, open new avenues for controlling and optimizing food production.

What unites these diverse approaches is their relevance to current challenges being faced. Ensuring sustainability, maintaining authenticity, and safeguarding traceability in complex supply chains are not merely technical issues—they are a concern of public trust and global food security. The contributions to this Special Issue therefore not only expand the scientific frontier but also provide practical strategies for industry and policy.

The first contribution by W. Wang et al. [Contribution List 1] investigates the kneading process in pasta production, emphasizing the critical impact of both under- and over-kneading on dough quality. The study introduces a novel and cost-effective approach that employs a current sensor to determine the optimal kneading time. During kneading, the dough’s tensile resistance gradually increases as key structural properties, such as the gluten network, develop. This progression is reflected in the rising load current of the mixing machine, which peaks before declining once over-kneading occurs. The authors clearly show that identifying this peak allows for precise determination of the optimal dough consistency, ultimately improving the quality of both the dough and the resulting pasta products.

The second paper, by S. Dirr and Ö. Karslioglu [Contribution List 2], focuses on stinging nettle (*Urtica dioica* L.) as a sustainable protein source, valued for its rich profile of bioactive compounds and medicinal properties. The study examines different cell disruption methods—such as pulsed electric fields and high-pressure homogenization—in combination with extraction techniques, including isoelectric precipitation, ultrafiltration, and salting-out, in order to optimize protein yield and evaluate effects on chlorophyll content. The results show that high-pressure homogenization coupled with isoelectric precipitation achieved the highest protein yield of 11.60%. In contrast, the combination of pulsed electric fields with ultrafiltration effectively reduced the chlorophyll content from 4781.41 µg/g in the raw leaves to 15.07 µg/g in the processed material. These findings demonstrate how innovative extraction strategies can enhance both the efficiency and sustainability of protein recovery from stinging nettle. In turn, they highlight the potential of this underutilized plant to contribute to food fortification and the development of functional ingredients.

The study by E. Jakubczyk et al. [Contribution List 3] investigated the impact of ultrasound pretreatment and drying temperature on the quality of dried Golden Delicious apples, with the aim of optimizing the process for reduced drying time and desirable product properties. Apple tissue was sonicated for 30–60 min and subsequently air-dried at 55–85 °C. Key parameters—including dry matter content, water activity, color, antioxidant activity, and hygroscopicity—were evaluated alongside drying kinetics. The results indicated that shorter sonication times increased the dry matter content and lowered water activity, while reduced drying temperatures limited water uptake and decreased hygroscopicity. Importantly, the tested conditions supported the preservation of bioactive compounds and enhanced antioxidant activity. Using a second-order polynomial model and variance analysis, the authors identified optimal conditions that balanced process efficiency with product quality: 30 min of sonication combined with drying at 80.9 °C.

The paper by M. Bekogianni et al. [Contribution List 4] explores the application of laser-induced breakdown spectroscopy (LIBS) and fluorescence spectroscopy for detecting adulteration of extra virgin olive oil (EVOO) with other edible oils (pomace, corn, sunflower, soybean) and for distinguishing EVOOs by geographical origin. Using the same set of samples, both techniques were directly compared. Spectroscopic data were processed with various machine learning algorithms, and the resulting predictive models were thoroughly evaluated for accuracy and robustness. The findings demonstrate that both LIBS and fluorescence spectroscopy perform highly in classification, confirming their potential for rapid, online, and in situ authentication of EVOO. Notably, LIBS proved particularly advantageous due to its faster operation.

The study by Y. Zhang et al. [Contribution List 5] examines mass transfer during ethanol pickling of dealuminated jellyfish, addressing the challenge of excessive aluminum in salted jellyfish and the resulting quality and shelf-life issues after dealumination. Ethanol, known for its protein-precipitating and bactericidal properties, was tested at varying concentrations to identify optimal processing conditions. The results show that the ethanol concentration and pickling time significantly influence mass transfer: higher concentrations reduced the overall mass while increasing ethanol uptake, with the most pronounced changes occurring in the early pickling stages. A 45% ethanol solution proved most effective, yielding texture properties comparable to those of edible jellyfish and offering suitable conditions for reprocessing. Moreover, the developed mass transfer model demonstrated a strong linear correlation with time, providing a practical tool for further process optimization.
